# Non-episodic angioedema with eosinophilia as a differential diagnosis of eosinophilia in young females^☆^

**DOI:** 10.1016/j.waojou.2024.100981

**Published:** 2024-10-24

**Authors:** Yura Ha, Min Ju Pyo, Ye Eun Hong, So Hye Nam, Woo-Jung Song, Hyouk-Soo Kwon, Tae-Bum Kim, Yoo Sook Cho, Ji-Hyang Lee

**Affiliations:** a Department of Medical Science, AMIST, Asan Medical Center, University of Ulsan College of Medicine, Seoul, Republic of Korea; b Division of Rheumatology, Department of Internal Medicine, Keimyung University, Dongsan Medical Center, Daegu, Republic of Korea; c Department of Allergy and Clinical Immunology, Asan Medical Center, University of Ulsan College of Medicine, Seoul, Republic of Korea; d Drug Safety Center, Seoul National University Hospital, Seoul, Republic of Korea

**Keywords:** Eosinophilia, Angioedema, Glucocorticoids, Asian, Females

## Abstract

**Background:**

Episodic angioedema with eosinophilia, also known as Gleich's syndrome, is a differential diagnosis in patients with recurrent angioedema with higher blood eosinophils. Meanwhile, less has been elucidated regarding non-episodic angioedema with eosinophilia (NEAE). This study aimed to examine the prevalence, clinical characteristics, and disease course of NEAE.

**Methods:**

By reviewing the electronic medical records, we identified patients with NEAE among those referred to allergy clinics due to eosinophilia from January 2021 to December 2023 at Asan Medical Center.

**Results:**

Among 687 patients with eosinophilia, 58 (8.4%) were diagnosed with and treated for NEAE. All patients were females, with a mean age of 31.79 years. The mean absolute blood eosinophil count was 4468.76 cells/μL. All patients reported symmetric angioedema of the lower legs, and 37 (63.8%) had additional angioedema of the upper arms. Twenty-five (43.1%) patients reported a preceding event prior to onset of angioedema. Systemic corticosteroids (mean total dose 1745 ± 508.49 mg) were prescribed to all patients, with a treatment duration of approximately 40 days to achieve resolution. Following the resolution of angioedema, 6 patients experienced persistent arthralgia, 1 developed chronic spontaneous urticaria, and 1 developed hypereosinophilic syndrome.

**Conclusions:**

NEAE is an essential differential diagnosis in young female patients with eosinophilia, particularly those presenting with symmetric peripheral angioedema.

## Introduction

Eosinophils generally comprise less than 5% of circulating leukocytes in healthy individuals. The level of blood eosinophils can increase on various occasions, even in the absence of specific triggers, which can lead to tissue damage and subsequent clinical manifestations.^1^ In the approach for patients with peripheral eosinophilia, physicians conduct diagnostic tests to identify the etiology of eosinophilia and examine the presence of organ damage induced by eosinophilic infiltration.^2^

Hypereosinophilic syndrome (HES) is diagnosed when there is sustained hypereosinophilia and organ damage/dysfunction due to tissue infiltration by eosinophils, excluding reactive eosinophilia and abnormal hematopoiesis. Patients with HES often experience weakness, fatigue, respiratory, gastrointestinal, cutaneous, and muscular symptoms dependent on involved organs.^3^ Episodic angioedema with eosinophilia (EAE), also known as Gleich syndrome, is featured with recurrent angioedema accompanied by eosinophilia, which may appear with fever, wheals, or weight gain.^4^ Owing to its distinct clinical features, EAE is considered a special variant of HES.^5^ In contrast to the proven therapeutic benefit of mepolizumab in HES, mepolizumab had failed to reduce the number of acute flares in patients with EAE despite reducing the number of eosinophils, suggesting the presence of pathomechanism not fully related to eosinophilic inflammation.^6^^,^^7^

Meanwhile, less has been elucidated regarding non-episodic angioedema with eosinophilia (NEAE). First reported in 1998 by Chikama and colleagues, NEAE is characterized by nonepisodic angioedema with eosinophilia, lack of fever, and no internal organ involvement.^8^ NEAE is considered a rare disease mostly affecting Asian females. Only a few case reports and small case series have been published to date, most of which were reported in Japan, Korea, and Thailand.^9^^,^^10^ Recently reported cases were related to COVID-19 and influenza vaccination.^11^, ^12^, ^13^, ^14^, ^15^ Due to its rarity, there are no standardized guidelines for the diagnosis or treatment of NEAE. Whether systemic corticosteroids (SCS) are required or if symptomatic treatment is sufficient remains controversial. In addition, there is considerable uncertainty regarding the epidemiology and clinical course of NEAE.

This study aimed to understand the prevalence, clinical characteristics, and disease course of NEAE and to identify the clinical features related to treatment outcomes by analyzing clinical data of patients with NEAE selected from those referred to an allergy clinic due to eosinophilia.

## Methods

### Study design and population

We conducted a retrospective study using electronic medical records (EMR) from the Asan Medical Center. Data of adult patients (aged 18 years and older) who visited allergy clinics for eosinophilia from January 2021 to December 2023 were extracted. NEAE was defined when the patient presented angioedema of extremities with documented eosinophilia (peripheral blood eosinophil ≥500 cells/μ). Patients with 1) eosinophilia of other etiologies, including drugs, underlying disease, parasite infection, or malignancy; 2) generalized edema; and 3) a diagnosis of inflammatory arthritis were excluded. The study was approved by the Institutional Review Board of Asan Medical Center (No. 2024-0861).

### Collected clinical data

Baseline demographic factors (age, smoking, and body mass index [BMI]), underlying allergic diseases (allergic rhinitis [AR], bronchial asthma [BA], atopic dermatitis [AD], and chronic idiopathic urticaria [CIU]), and clinical features were collected from medical records. In terms of angioedema, preceding events, affected areas, symptom onset, coexisting systemic or cutaneous symptoms, and clinic visits during management were included. Unusual events that had occurred up to the previous month of angioedema as reported by the patients were considered preceding events. Laboratory measurements included white blood cell counts, eosinophils, total IgE, erythrocyte sedimentation rate (ESR), C-reactive protein (CRP), lactate dehydrogenase (LDH), D-dimer, antinuclear antibody (ANA), and antineutrophil cytoplasmic antibody (ANCA). The degree of eosinophilia was categorized as mild, (500–1500 cells/μL), moderate (1500–5000 cells/μL), or severe (greater than 5000 cells/μL). The cutoff levels of total IgE, ESR, CPR, LDH, and d-dimer were 100 U/mL, 20 mm/h, 0.6 mg/dL, 250 IU/L, and 0.5 μg/mL FEU, respectively. Additional tests for evaluating the etiology of eosinophilia and angioedema and their results were also reviewed. For each eligible patient with NEAE, we reviewed the medical records to identify the prescription of medications, including the duration and dose of SCS and additional medications. The sequelae after the resolution of angioedema were also reviewed.

### Statistical analysis

Continuous variables are described as mean ± standard deviation (SD), and categorical variables are expressed as numbers (percentages). Student t or χ^2^ test was used for comparisons between groups. Spearman's correlation was used to estimate the correlation between clinical variables. Multivariate analyses were conducted to identify factors associated with the required dose of corticosteroids and the total duration of treatment. All statistical analyses were performed using SPSS software (version 22.0, IBM Corporation, Armonk, NY, USA). Statistical significance was set at P < 0.05.

## Results

**Fig. 1:**
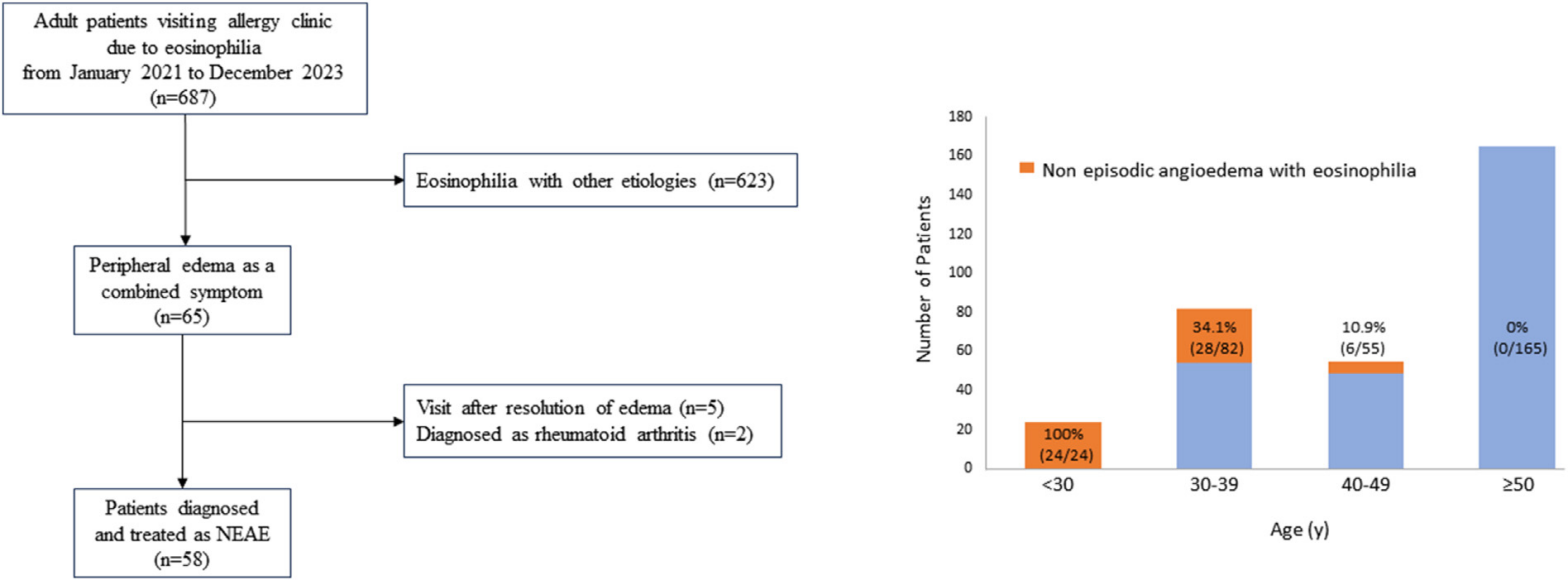
Flow chart of selection of eligible patients and proportion of patients with NEAE. (a) Flow chart of selection of patients diagnosed and treated with NEAE (b) Proportion of patients with NEAE among the patients with eosinophilia. NEAE, non-episodic angioedema with eosinophilia

From January 2022 to December 2023, a total of 687 patients were referred to the allergy clinic for eosinophilia. Of these, 65 had peripheral angioedema. However, 5 patients were asymptomatic at the time of clinic visit, and 2 were diagnosed with rheumatoid arthritis (RA). Finally, 58 patients diagnosed with and treated for NEAE were included in the analysis. All patients with NEAE were female (Fig. 1a). The prevalence of NEAE among women with eosinophilia differed significantly by age group, with the highest prevalence in patients younger than 30 years (100%, 24/24), followed by those aged in their thirties (34.1%, 28/82), 40s (10.9%, 6/55), and no cases in women aged 50 years or older (*p* < 0.001) (Fig. 1b).

The mean age of the patients was 31.79 years (Table 1). None of the patients were smokers, and their mean BMI was 22.29 kg/m^2^. Fifteen patients (25.9%) reported coexisting allergic conditions, including AR (10/58, 17.2%), CIU (4/58, 6.9%), BA (3/58, 5.2%), and AD (3/58, 5.2%). Preceding events were reported by 25 patients (48%) within 1 month of angioedema onset. Vaccination (11/58, 19%) was the most common event, followed by inflammatory conditions (6/58, 10.3%), vigorous exercise (3/58, 5.2%), botulinum toxin injection (2/58, 3.4%), emotional stress (2/58, 3.4%), and acupuncture (1/58, 1.7%). The 11 preceding vaccinations included 9 COVID-19 vaccinations and 2 influenza vaccinations. The inflammatory conditions included 3 cases of plantar fasciitis and 1 case each of cystitis, COVID-19 infection, and upper respiratory tract infection.

**Table 1:** Clinical characteristics of patients with NEAE.

Variables	Results
Age, years	31.79 ± 6.03
Sex, females	58 (100)
BMI, kg/m^2^	22.29 ± 4.18
Never smoker	58 (100)
Allergic diseases	
Allergic rhinitis	10 (17.2)
Chronic idiopathic urticaria	4 (6.9)
Bronchial asthma	3 (5.2)
Atopic dermatitis	3 (5.2)
Presence of preceding events	25 (43.1)
Vaccination	11 (19.0)
Inflammation	6 (10.3)
Vigorous exercise	3 (5.2)
Injection of botulinum toxin	2 (3.4)
Emotional stress	2 (3.4)
Acupuncture	1 (1.7)
Symptom onset, weeks	3.16 ± 1.68
Affected area	
Lower extremities, symmetric	58 (100)
Upper and lower extremities, symmetric	37 (63.8)
Combined symptoms	
Pain	53 (91.4)
Pruritus	24 (41.4)
Urticaria	15 (25.9)
Maculopapular rash	5 (8.6)
Fatigue	4 (6.9)

BMI, body mass index

**Fig. 2:**
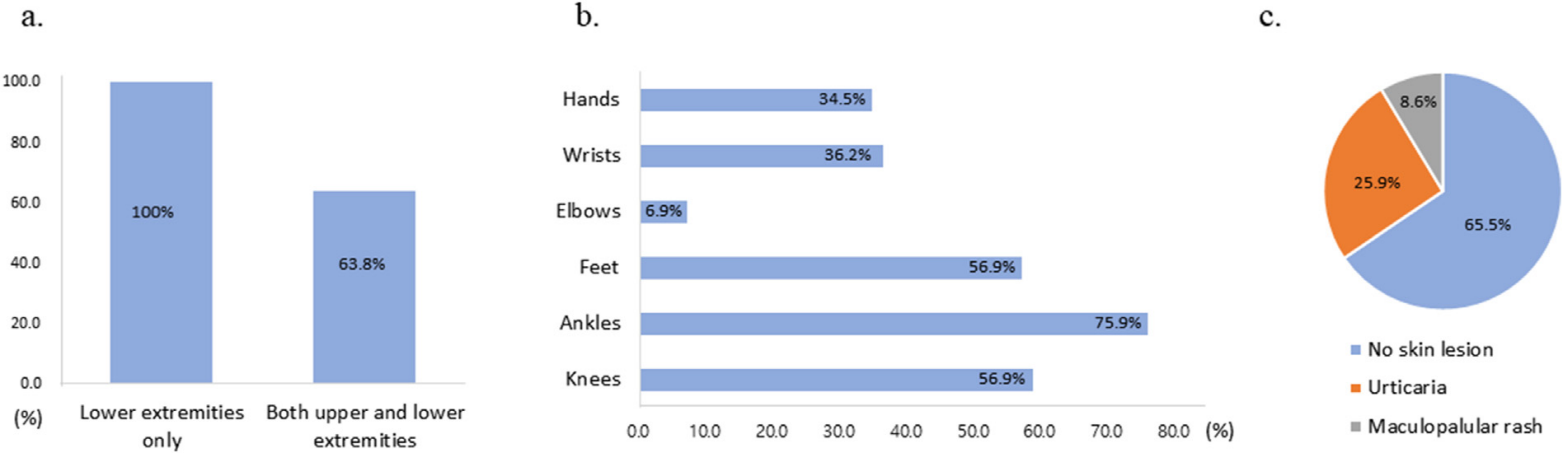
Clinical characteristics of eosinophilic angioedema. (a) Proportion of affected extremities in patients with NEAE (b) Distribution of affected areas with angioedema (c) Cutaneous manifestations in patients with NEAE. NEAE, non-episodic angioedema with eosinophilia

The mean symptom duration before diagnosis was 3.16 weeks. All patients had symmetric angioedema in the lower extremities, and 37 patients (63.8%) experienced simultaneous angioedema in both the upper and lower extremities (Fig. 2a). In the upper extremities, the wrists were most frequently affected (36.2%), followed by the hands (34.5%) and elbows (6.9%). In the lower extremities, the most commonly affected areas were the ankles (75.9%), followed by the feet and knees (56.9% each) (Fig. 2b). Coexisting cutaneous manifestations were observed in 20 patients (34.5%) (urticaria in 15 patients [25.9%] and maculopapular rash in 5 patients [8.6%]) (Fig. 2c). Most patients (53/58, 91.4%) complained of pain, and 24 (41.4%) reported pruritus (Table 1).

**Fig. 3:**
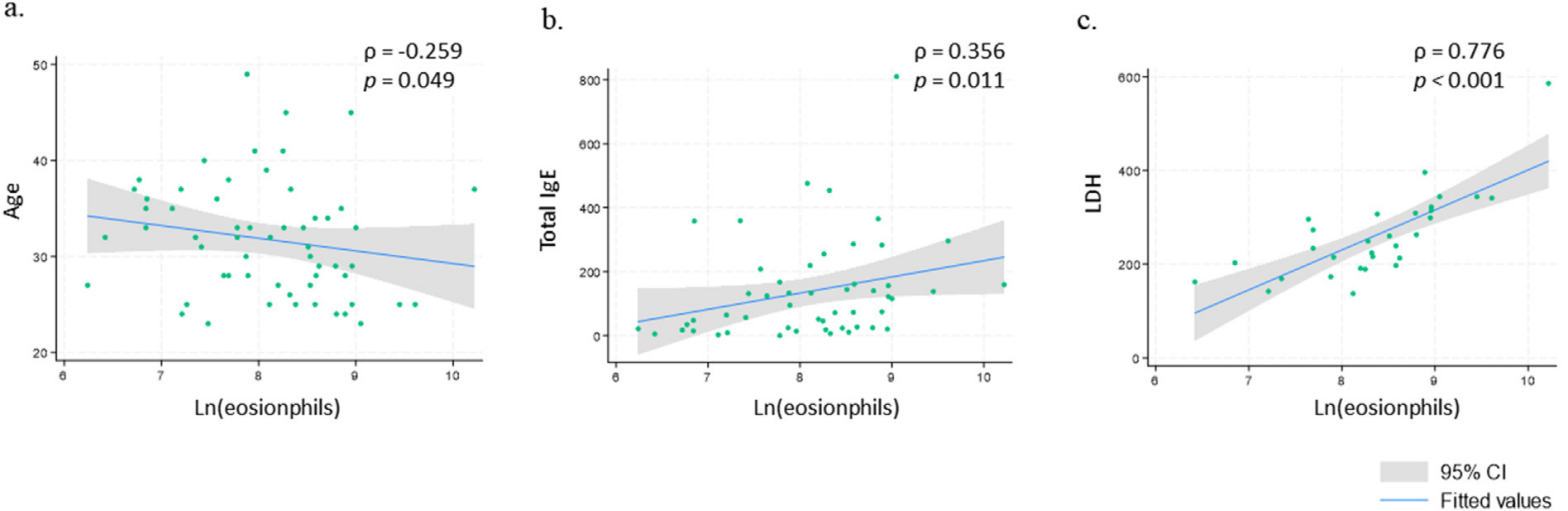
Relationships between clinical variables in NEAE. Correlation between blood eosinophils and (a) age, (b) total IgE, as well as (c) LDH. NEAE, non-episodic angioedema with eosinophilia; LDH, lactate dehydrogenase

In terms of laboratory findings, the mean absolute eosinophil count was 4468.76 cells/μL. Elevated levels of total IgE, ESR, CRP, LDH, and D-dimer were observed in 48.1%, 12.1%, 17.5%, 46.7%, and 75% of patients tested, respectively (Table 2). Among the 38 patients who underwent ANCA testing, all had negative results, while ANA tests were positive in 18% (6/33). A significant negative correlation was found between patient age and eosinophil count (ρ = −0.259, *p* = 0.049) (Fig. 3a). Between the laboratory data, blood eosinophil levels were positively correlated with total IgE (ρ = 0.356, *p* = 0.011) and LDH (ρ = 0.776, *p* < 0.001) (Fig. 3b and c).

**Table 2:** Laboratory findings of NEAE.

Laboratory data	Results
WBC, × 10^3^/mm^3^	11.94 ± 4.92
Eosinophils, cells/mm^3^	4468.76 ± 4227.01
500 ≤ Eosinophils < 1500	11 (19.0)
1500 ≤ Eosinophils < 5000	27 (46.6)
5000 ≤ Eosinophils	20 (34.5)
Total IgE, U/mL (n = 52)	138.08 ± 155.97
Elevated total IgE, ≥100U/mL	25 (48.1)
ESR, mm/hr (n = 33)	9.48 ± 8.69
Elevated ESR, ≥20 mm/h	4 (12.1)
CRP, mg/dL (n = 40)	0.50 ± 0.91
Elevated CRP, ≥0.6 mg/dL	7 (17.5)
LDH, IU/L (n = 30)	260.27 ± 91.00
Elevated LD, ≥250 IU/L	14 (46.7)
D-dimer, ug/ml FEU (n = 28)	1.35 ± 2.90
Elevated D-dimer, ≥0.5 μg/ml FEU	21 (75)
Positive ANA (n = 33)	6 (18.2)
Positive ANCA (n = 38)	0 (0)

BMI, body mass index; WBC, white blood cell; ESR, erythrocyte sedimentation rate; CRP, C-reactive protein; LDH, lactate dehydrogenase; ANA, antinuclear antibody; ANCA, Antineutrophil Cytoplasmic Antibodies

During the diagnostic workup and treatment, 27 (46.6%) patients visited specialists in addition to allergists (Table 3). Rheumatologists were the most frequently consulted (14/58, 24.1%), followed by orthopedic surgeons (9/58, 15.5%) and hematologists (6/58, 10.3%). Twenty-three patients (39.7%) underwent additional tests to rule out rheumatoid arthritis, hypereosinophilic syndrome, or primary hypereosinophilia due to abnormal bone marrow (BM) function. Additional tests included joint ultrasonography (12/58, 20.7%), chest and abdominal computed tomography (CT) (10/58, 17.2%), lower extremity CT (3/58, 5.2%), lower extremity magnetic resonance imaging (MRI) (2/58, 3.4%), and BM biopsy (2/58, 3.4%). Imaging modalities evaluating the joints, including ultrasonography, MRI, and CT, showed joint subcutaneous and soft-tissue edema. A small amount of joint effusion was observed in 4 cases (4/15, 26.7%). None of the patients exhibited synovial proliferation, suggesting rheumatoid arthritis (RA). Additionally, no evidence of internal organ involvement or hematopoietic abnormalities was detected on CT or BM biopsy.

**Table 3:** Management and treatment outcome of NEAE.

Variables	Results
Clinics visited for management	
Rheumatology	14 (24.1)
Orthopedics	9 (15.5)
Hematology	6 (10.3)
Rehabilitation medicine	3 (5.2)
Infection	2 (3.4)
Dermatology	1 (1.7)
Additional diagnostic tests	23 (39.7)
Joint ultrasonography	12 (20.7)
CT, chest	10 (17.2)
CT, abdomen	10 (17.2)
CT, low extremities	3 (5.2)
MRI, low extremities	2 (3.4)
Bone marrow biopsy	2 (3.4)
Duration of treatment, days	41.83 ± 33.32
Prescribed medication	
Systemic corticosteroids	58 (100)
H1RAs	20 (34.5)
NSAIDs	6 (10.3)
Reslizumab	2 (3.4)
LTRA	1 (1.7)
Total dose of SCS, mg	1745 ± 508.49
Presence of sequelae	8 (13.8)
Types of sequelae	
Arthralgia	6 (10.3)
Chronic spontaneous urticaria	1 (1.7)
Hypereosinophilic syndrome	1 (1.7)

CT, computed tomography; MRI, magnetic resonance image; H1RAs, histamine-1 receptor antagonists; NSAIDs, non-steroidal anti-inflammatory drugs; LTRA, leukotriene receptor antagonist; SCS, systemic corticosteroids

All patients were treated with SCS (mean total dose of 1745 ± 508.49 mg in prednisolone equivalent), and the mean duration of treatment was 41.83 days. The SCS was gradually tapered or discontinued based on symptom severity. We have noted that twelve patients (12/58, 20.7%) required an increase in corticosteroid dosage during the tapering phase, indicating fluctuations in disease activity. Additional medications included a histamine-1-receptor antagonist (34.5%), nonsteroidal anti-inflammatory drugs (10.3%) and leukotriene receptor antagonists (1.7%). Two patients received a single intravenous dose of reslizumab (100 mg). After the resolution of the angioedema, 6 (10.3%) patients experienced persistent arthralgia, 1 developed chronic spontaneous urticaria, and 1 developed HES.

Next, we investigated the factors affecting the total dose of corticosteroids and duration of treatment (Table 4). Multivariate analysis revealed that involvement of both upper and low extremities (β = 0.256, *p* = 0.045) and greater than moderate eosinophilia (blood eosinophils ≥1500/μL) (β = 0.326, *p* = 0.018) were associated with a higher total corticosteroid dose. However, age, BMI, underlying allergic disease, preceding events, and cutaneous symptoms were not associated with the total dose of corticosteroids. No demographic or clinical characteristics were associated with the total duration of treatment.

**Table 4:** Factors related to treatment duration/total dose of SCS.

Variables	Total dose of corticosteroids		Total duration of treatment	
	β-coefficient	P value	β-coefficient	P value
Age	0.187	0.146	−0.359	0.721
BMI	0.120	0.351	−0.706	0.484
Onset of symptom	0.025	0.850	−0.311	0.757
Presence of allergic disease (ref. absence)	−0.140	0.277	0.102	0.919
Presence of preceding events (ref. absence)	0.100	0.435	1.833	0.073
Upper and lower extremities (ref. only lower extremities)	0.256	0.045	1.324	0.192
Presence of cutaneous symptoms (ref. absence)	−0.179	0.165	−1.464	0.149
Blood eosinophils ≥1500 (ref. <1500)	0.326	0.018	1.818	0.075

BMI, body mass index

## Discussion

In this study, we described the detailed clinical manifestations and disease course of NEAE. Among 687 patients referred to the allergy clinic for eosinophilia, 8.4% (58/687) were diagnosed with NEAE. NEAE predominantly affected young females in their 20s and 30s, with nearly half of these patients reporting preceding events. All patients presented with symmetric angioedema in the lower legs, and one-third also had cutaneous manifestations. All patients received SCS to control symptoms for approximately 40 days. Involvement of both upper and lower extremities and greater than moderate eosinophilia (≥1500/μL) were significantly associated with a higher total SCS dose. To the best of our knowledge, this is the largest study to date analyzing the clinical features of NEAE in detail.

In contrast to EAE, which is characterized by cyclic angioedema lasting about 5 to 7 days followed by asymptomatic periods every 3 to 8 weeks over the course of several years, NEAE presents with persistent symptoms lasting for several weeks without recurrence once resolved.^16^ NEAE differs from typical angioedema, which usually affects the face, lips, larynx, and tongue with an asymmetric distribution. However, it shares the characteristics of non-pitting subcutaneous edema with other forms of angioedema.^17^ In NEAE, symmetric angioedema of the lower extremities is a hallmark, with the distal parts of the limbs more affected than the proximal parts. This pattern of distribution is similar to that of RA, which is characterized by arthritis of the hands, wrists, and feet.^18^ In our analysis, 2 patients aged in their forties who were initially suspected of having NEAE were diagnosed with RA and thus excluded from the analysis. Rheumatoid factor and anti-CCP antibody tests were performed in 19 and 15 patients, respectively, all results were negative. Although mild eosinophilia can occur in RA, severe hypereosinophilia in young females is more indicative of NEAE.^19^

Although NEAE is considered a rare condition, our findings demonstrated that approximately 8% of the patients visiting tertiary hospital for eosinophilia were diagnosed with NEAE. This contrasts with a previous retrospective study conducted in 3 Korean hospitals, which identified 10 patients over 6 years from 2007 to 2012. The higher number of patients in our study could suggest either underdiagnosis in the past or a true increase of NEAE incidence among Koreans, although its precise prevalence in the general population is yet to be estimated.^10^ Although the massive nationwide COVID-19 vaccinations may have affected the increased incidence, NEAE following the COVID-19 vaccination comprises only 15.5% of total cases.

Notably, nearly half of the patients reported an event prior to the development of peripheral angioedema. Most cases involve vaccinations, inflammatory conditions, or injections. There was no correlation between the injection site and the location of angioedema, implicating NEAE as a part of a systemic immune reaction. Three patients were treated for plantar fasciitis, which may have been the initial manifestation of NEAE. There could be heterogeneity in the development of NEAE, which could be either a reactive response to external stimuli or a natural development. However, no difference in clinical manifestations or outcomes were observed between patients with or without preceding events. The presence of positive ANA in some patients may indicate underlying autoimmune reactions in certain cases.^20^

Approximately half of the patients visited multiple specialists during diagnosis and treatment. The duration of symptoms before diagnosis was up to 3 weeks. This highlights the need to increase awareness of NEAE among both clinicians and patients. In clinical practice, angioedema could be confused with joint swelling suggestive of arthritis, leading many patients to visit rheumatology or orthopedic clinics. Diagnosing NEAE is difficult without blood tests, which are not routinely conducted in young patients with leg pain in the first place, contributing to delayed diagnosis. In addition, because the level of blood eosinophils is often strikingly elevated, some clinicians perform CT scan or BM biopsy to exclude organ damage and hematological malignancies. Given the good treatment response to SCS, early suspicion and diagnosis of NEAE are required to avoid unnecessary diagnostic tests and clinic visits.

Another concern raised by this study is the substantial dose of SCS required to manage NEAE. Exposure to even small doses of SCS is associated with an increased risk of adverse events. The risk of adverse events increases when the accumulated dose exceeds 500 mg, which further increases to 1000 mg.^21^ The total SCS dose for treating NEAE was even greater than the dose required to treat drug reactions with eosinophilia and systemic symptoms syndrome, a severe cutaneous adverse reaction.^22^ Since the majority of the patients are young females, we should consider potential adverse effects of SCS. Some cases of NEAE were reported, which resolved without medication or with symptomatic treatment alone.^13^^,^^23^ In our data, among 5 patients suspected of having NEAE who visited the clinic after the resolution of angioedema, 2 were treated with NSAIDs, and 1 resolved without medication. Further research is needed to identify which patients truly require SCS and to consider steroid-sparing strategies or early use of biologics to minimize corticosteroid toxicity.

Eight patients reported some type of sequelae. The most common sequela was arthralgia without angioedema, which resolved with symptomatic treatment. Follow-up is recommended to manage potential sequelae. Given the development of CIU and HES after the resolution of angioedema, NEAE could be an initial manifestation of an autoimmune or systemic eosinophilic disorder.

This study has several limitations. First, since the study was conducted at a single tertiary hospital, the findings may not be generalizable to all NEAE cases. Second, the evaluation of sequelae was based on medical records, and the follow-up period varied among patients. Third, the preceding events collected solely relied on the participants’ memories. In addition, there is no feasible method to evaluate the causality. Finally, as all patients received SCS, the necessity or superiority of SCS compared to other treatments for NEAE was difficult to assess. The natural course of NEAE without intervention remains inconclusive. Despite these limitations, our study demonstrated the clinical characteristics of NEAE in detail.

In conclusion, NEAE is a common cause of eosinophilia in young females. For female patients presenting with hypereosinophilia with peripheral angioedema, the diagnosis of NEAE and initiation of SCS should be considered before invasive diagnostic processes. Further research is required to elucidate the pathogenesis of NEAE and establish standardized diagnostic and therapeutic guidelines.

## Abbreviations

HES, hypereosinophilic syndrome; EAE, episodic angioedema with eosinophilia; NEAE, non-episodic angioedema with eosinophilia; SCS, systemic corticosteroids; EMR, electronic medical record; AR, allergic rhinitis; BA, bronchial asthma; AD, atopic dermatitis; CIU, chronic idiopathic urticaria; ESR, erythrocyte sedimentation rate; CRP, C-reactive protein; LDH, lactate dehydrogenase; ANA, antinuclear antibody; ANCA, antineutrophil cytoplasmic antibody; RA, rheumatoid arthritis; BMI, body mass index; CT, computed tomography; MRI, magnetic resonance image; BM, bone marrow.

## Availability of data and materials

Data are available from the corresponding author on reasonable request.

## Author's contributions

JHL and YSC designed the study and wrote the manuscript. MJP, YEH, WJS, HSK, and TBK contributed to data collection. SHN and JHL performed the statistical analysis and interpretation of the results. All authors read and approved the final manuscript.

## Ethics approval and consent to participate

The study was approved by the Institutional Review Board of Asan Medical Center (No. 2024-0861).

## Consent for publication

All authors approved the final version of manuscript for submission.

## Declaration of competing interest

The authors declare that there is no conflict of interest to declare.
